# Development of a patient-reported outcome measure of digital health literacy for chronic patients: results of a French international online Delphi study

**DOI:** 10.1186/s12912-023-01633-x

**Published:** 2023-12-14

**Authors:** Carole Délétroz, Claudio Del Grande, Samira Amil, Patrick Bodenmann, Marie-Pierre Gagnon, Maxime Sasseville

**Affiliations:** 1 Doctoral Candidate, Faculty of Nursing Sciences, Université Laval, Canada and School of Health Sciences (HESAV), University of Applied Sciences and Arts Western Switzerland, Avenue de Beaumont 21, 1011 Lausanne, Switzerland; 2 Doctoral Candidate, School of Public Health, University of Montreal and Research Associate, Health Innovation and Evaluation Hub, University of Montreal Hospital Research Centre, 850 Rue St-Denis, Montréal, Québec QC H2X 0A9 Canada; 3 Doctoral Candidate, Centre Nutrition, Santé Et Société (NUTRISS)-INAF, Université Laval, Québec, Canada and VITAM - Centre de Recherche en Santé Durable and Unité de Soutien Au Système de Santé Apprenant du Québec, Québec, Canada; 4 Department of Vulnerabilities and Social Medicine, Unisanté, Lausanne and Faculty of Biology and Medicine, Vice-Dean Teaching and Diversity, University of Lausanne, Rue du Bugnon 44, 1011 Lausanne, Switzerland; 5 Faculty of Nursing Sciences, Université Laval, 2325 Rue de L’Université, Québec, QC G1V 0A6 Canada

**Keywords:** Digital health literacy, Health literacy, Delphi technique, Patient reported outcome measure, Validation studies, Chronic disease

## Abstract

**Background:**

A psychometrically robust patient-reported outcome measure (PROM) to assess digital health literacy for chronic patients is needed in the context of digital health. We defined measurement constructs for a new PROM in previous studies using a systematic review, a qualitative description of constructs from patients, health professionals and an item pool identification process. This study aimed to evaluate the content validity of a digital health literacy PROM for chronic patients using an e-Delphi technique.

**Methods:**

An international three-round online Delphi (*e*-Delphi) study was conducted among a francophone expert panel gathering academics, clinicians and patient partners. These experts rated the relevance, improvability, and self-ratability of each construct (n = 5) and items (n = 14) of the preliminary version of the PROM on a 5-point Likert scale. Consensus attainment was defined as strong if ≥ 70% panelists agree or strongly agree. A qualitative analysis of comments was carried out to describe personal coping strategies in healthcare expressed by the panel. Qualitative results were presented using a conceptually clustered matrix.

**Results:**

Thirty-four experts completed the study (with 10% attrition at the second round and 5% at the third round). The panel included mostly nurses working in clinical practice and academics from nursing science, medicine, public health background and patient partners. Five items were excluded, and one question was added during the consensus attainment process. Qualitative comments describing the panel view of coping strategies in healthcare were analysed. Results showed two important themes that underpin most of personal coping strategies related to using information and communications technologies: 1) questionable patient capacity to assess digital health literacy, 2) digital devices as a factor influencing patient and care.

**Conclusion:**

Consensus was reached on the relevance, improvability, and self-ratability of 5 constructs and 11 items for a digital health literacy PROM. Evaluation of e-health programs requires validated measurement of digital health literacy including the empowerment construct. This new PROM appears as a relevant tool, but requires further validation.

**Supplementary Information:**

The online version contains supplementary material available at 10.1186/s12912-023-01633-x.

## Background

Digital health literacy (DHL) is a concept that aims to improve competencies of patients and communities who are facing problems associated with processing health information from digital devices every day [1]. DHL is defined as “the ability to search, find, understand, evaluate health information from electronic sources and apply the knowledge gained to address or solve a health problem” [2, 3]. It has been estimated that 75.8% of patients have low or problematic DHL in the German population, 72% in the Swiss population and 52.7% in the Portuguese population [4]. De Gani and colleagues point out that in Switzerland, low DHL particularly affects the elderly, people living with a chronic disease, or living in financial deprivation, and those having difficulties with the local language or receiving little social support [5]. People with high DHL report better self-perceived health, are less likely to have chronic diseases or health problems and feel less restricted in their activities if they do suffer from chronic diseases or health problems [6, 7]. Healthier people could also probably have better DHL, so it is essential for nurses and other health professionals taking care of chronic patients to measure DHL [8, 9].

Nurses are uniquely positioned to initiate and facilitate DHL evaluation in clinical practice when using any forms of information or communication technologies (ICTs), such as telehealth interventions [10]. Furthermore, there are clinical recommendations for the use of hybrid models that include in person and virtual care, with the aim of facilitating or maximizing the quality and effectiveness of patient care [11, 12]. Efforts have been made, therefore, to specify the measurement needed to improve patients’ DHL. Several tools were developed to assess DHL, such as the eHealth Literacy Scale or eHEALS [13], the eHealth Literacy Questionnaire (eHLQ) [14], the Digital Health Literacy Instrument (DHLI) [15], and HL-DIGI of M-POHL 2019 [16].

Existing tools have been criticized for being too long, of poorly reported psychometric properties [17–19]. First, existing tools don’t account for patients’ abilities to interact about adaptation coping process with health professionals (e.g., typing, search information, share opinion and emotion) through digital devices [17], which is indispensable when using e-health. In addition, common techniques to elicit information from adults are questionnaires or online questionnaires [17, 19]. It should also be pointed out that one key limiting factor in enabling patients to engage with digital resources is DHL [18]. So, it is an important methodological point to get input from the target population and clinicians to provide a clear definition of the concept DHL to be measured. Considering input of patient and health professionals enables to have a definition that “is a statement of an understanding of the construct DHL to be measured” in clinical practice, and of measurements constructs such as digital literacy, information literacy [20]. By better understanding the level of DHL of individuals, it is possible to identify the needs of specific groups in order to develop appropriate information or education interventions and ensure equitable access to healthcare for a broader public.

**Fig. 1:**
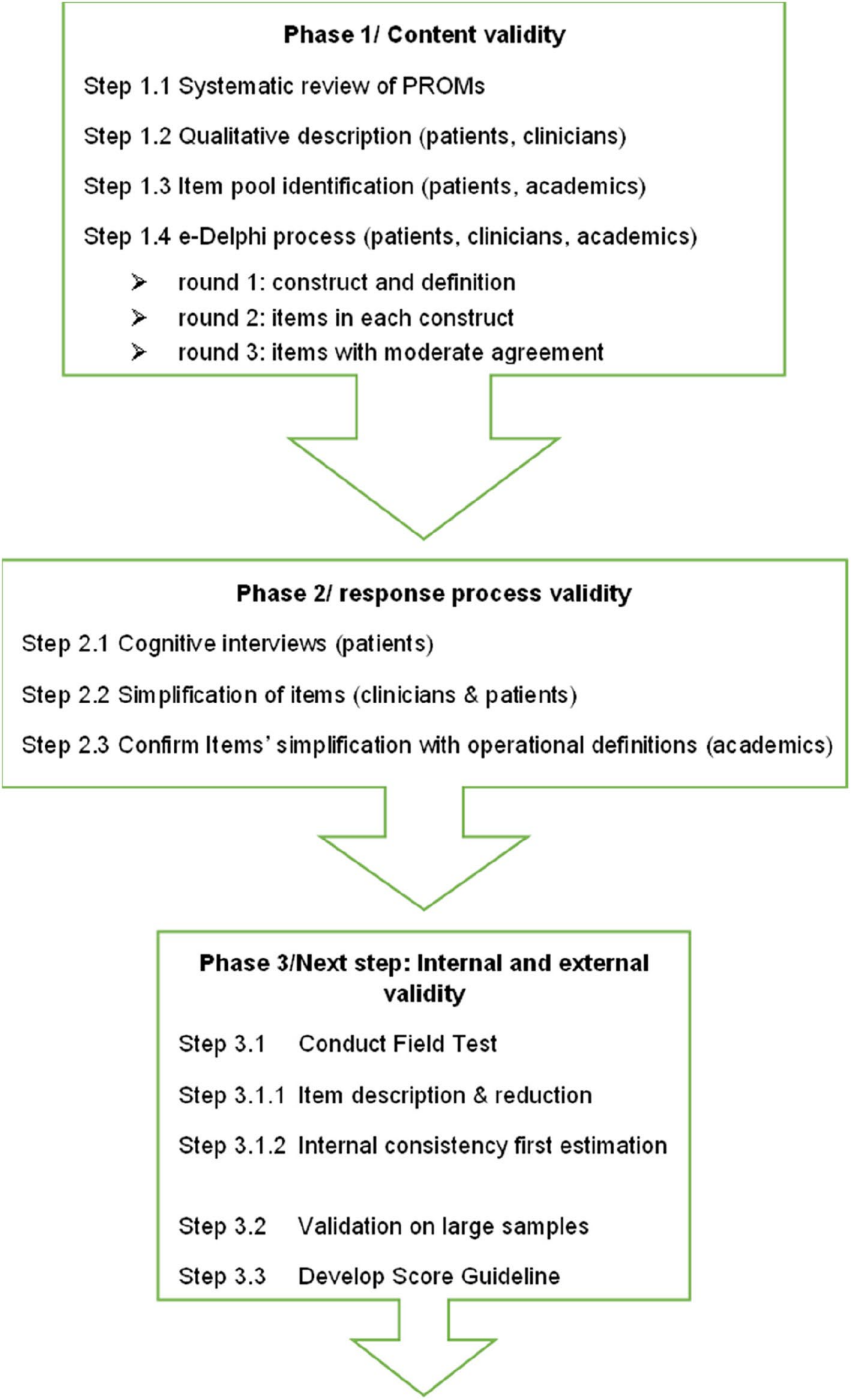
Flow-chart of the PROM development process

A comprehensive, psychometrically robust patient-reported outcome measure (PROM) to assess DHL for personal health among chronic patients does not yet exist. PROMs are defined as standardized, validated questionnaires (also called instruments) related to patient’s health status, that are completed by patients [21, 22]. This multi-phase research project aimed to develop a DHL PROM for chronic patients following the COnsensus-based Standards for the selection of health Measurement INstruments (COSMIN) recommendations [19]. Using the Roy adaptation model as a framework [23, 24], we previously conducted a qualitative exploration of chronic patients and professionals (nurses and doctors) understanding and definition of DHL when using ICTs in healthcare. These findings, combined with those of a systematic review of existing DHL measures, informed the development of a preliminary PROM [25]. This study aimed to evaluate the content validity of a digital health literacy PROM for chronic patients using an e-Delphi technique (step 1.4 in Fig. 1).

## Methods

### Preliminary development (Phase 1, step 1.1 to 1.3)

Following the COSMIN’s recommendations [26], the first step – conceptual framework – addressed the need for a detailed definition of the constructs of DHL and chronic patients in the context of use of ICTs. A systematic review of DHL PROMs [17] and a qualitative analysis of outcome constructs resulting from DHL’s skills that are relevant for patients and clinicians were performed (steps 1.1 and 1.2 in Fig. 1).

The item pool identification process for each defined measurement construct described as relevant was extracted from the systematic review. This process allowed to list all measurement instruments and a set of relevant items that can be used to measure digital health literacy [25]. So, each item from each retained PROM was extracted and listed (steps 1.1 and 1.2 in Fig. 1). A first evaluation process on the initial pool of items (*n* = 67) was done with 4 patient partners of our research committee with different levels of digital health literacy. The items were then confirmed following the cross check of the qualitative results of the previous step (step 1.2).

A total of 27 items were retained from this first screening. Content for each item was extracted from existing measures and missing items were formulated. Then, a consensus process by three researchers was made for each domain and items according to De Walt's criteria on consistence, clarity, applicability and not confusing of items (27, p.4) (steps 1.3 in Fig. 1). Finally, 11 items based on an existing questionnaire, DHLI [15], were translated from English to French using the WHODAS 2.0 Translation package [28–30] and three items were formulated by patient partners members of our research committee. A formal request for the use of DHLI’s questions was sent to the authors. This PROM aimed at measuring self-reported improvement in abilities when using personal health information from ICTs.

### e-Delphi study (Phase 1, step 1.4)

We used an e-Delphi approach to conduct this study. This manuscript is written in accordance with the CREDES guideline for the reporting of Delphi studies [31]. We followed recommendations to define outcomes criteria indicators [32, 33] (see Table 1). The Delphi method is a structured process whose components (anonymity, iteration, controlled feedback, and statistical aggregation) aim to improve the pooling of experts’ opinions [34, 35]. More specifically, the Delphi method structures the communication process through rounds of questionnaires, eliminating or reducing some of the problems often present when experts are directly confronted in face-to-face discussions (*e.g.*, dominating personalities, time constraints) to obtain a more reliable group opinion and to identify areas of consensus or dissent [36–38]. Given the complex professional and caring relationships between primary care clinicians (doctors, nurses, etc.) and patients, as well as the potential tensions between clinical, experiential and academic expertise, we thought the Delphi approach offered a safe and rigorous communication’s format. Furthermore, experts that are geographically distant could take part in the asynchronous communication process due to the accessibility of the questionnaire online.

**Table 1:** Definitions, statistical measures of consensus and outcome criteria indicators

	Definition	Statistics	Round 1	Round 2	Round 3
Consensus	The degree (measure) to which a group of experts shares the same opinion (30,p.1525)	Percentage of agreement Interquartile range (IQR)	≥ 70%	≥ 70% IQR < 1	≥ 70%
Outcome criteria indicators	If agreement ≥ 70% (proportion of experts responding they agree or strongly agree), item or construct accepted If agreement between 50 and 69%, item or construct reassessed in the next round If agreement ≤ 49%, item or construct rejected		Construct	Items	Items reassessed and reworded items

In this study, we used a three-round online Delphi (e-Delphi) process to assess the content validity of the constructs and items of a preliminary PROM by assessing experts’ agreement on relevance (R), improvability (I), and self-ratability (S-R). Expert assessed each construct or item according to three criteria [39]: 1) relevance: the construct or item is relevant to assess digital health literacy; 2) improvability: measurement of construct or item is improvable by a clinical intervention; 3) self-ratability: the construct or item is self-ratable by patients.

Because the first qualitative exploration was made in French in Switzerland, methods in this study used a French version of the questionnaire while this paper is reported in English.

### Expert eligibility, recruitment of the panel

#### Panel size

There is no universal guide to sample size calculation in Delphi studies. According to Belton et al. a minimum of 5–20 should be used for Delphi studies [40]. Furthermore, a personalized approach is essential when communicating with participants to sustain their engagement and reduce dropouts [38]. According to a systematic review [41] of 80 Delphi studies in healthcare, the median number of Delphi panel members is 17 (interquartile range = 11–31). Considering the heterogeneity of the expert panel pursued in our study (patients, academics, clinicians), we aimed to recruit around 40 participants to obtain a manageable panel of about twice that median size assuming a 15% loss to follow up. No specific ratios per type of expert have been pre-defined.

### Participant recruitment

A purposive sampling method was used. Experts from three distinct groups were recruited in French speaking countries worldwide if they were aged 18 or above, able to read and write in French and willing to participate:

- Academics: eligible academics had to have 2 peer-reviewed publications focused on e-health, digital health literacy or self-reported measures indexed in Pubmed. They were identified through existing researcher networks and Universities.
- Clinicians: Healthcare professionals who used ICTs: technological resources including health websites, health apps or connected objects, telemedicine or telehealth in clinical practice with chronic patients (*e.g.*, nurses, doctors). Eligible participants had to have a relevant qualification (*e.g*., medical, nursing degree) and over 6 months experience in clinical practice, including working with low literacy populations. They were identified through existing professional networks in the research committee.
- Patient partners: These participants were purposively selected based on their known expertise in the use of ICTs and chronic condition/s from official registered patient partners’ associations in Belgium, France, Canada, and Switzerland. They had to have an actual utilization or to have used technological resources, either directly or with the support of a relative.

The research project was approved by the Ethics Committee of Laval University (CERUL) in Canada (2021–057,10^th^ December 2021). Participant recruitment and data collection took place from January 2022 to April 2022.

### e-Delphi process

Online questionnaires content was based on the preliminary development process (Fig. 1). All rounds were completed electronically and anonymously using Research Electronic Data Capture (REDCap) software [42, 43]. We planned to stop the Delphi process after three rounds, because we thought that additional rounds were unlikely to introduce significant changes and were not worth the risk of increasing attrition rates due to the repetitive nature of the exercise [44].

All online questionnaires were elaborated and pretested with a member from the research team and one patient partner, to ensure readability and clarity. The first questionnaire in round one was used to assess agreement level with DHL constructs. Statements (items) within DHL constructs were rated in rounds 2 and 3. All questionnaires used a 5-point Likert scale (from 1 = strongly disagree to 5 = strongly agree) and optional open text comment sections. An example of the assessed statements is presented in Table 2.

**Table 2:** Sample of survey questions used for empowerment construct

	Criteria	e-Delphi questions content
Round 1 Construct: empowerment	Relevance	Empowerment is a relevant area for assessing patients' digital health literacy
	Improvability	Empowerment is influenced by interventions (*e.g.* training, support, environmental modifications, incentives etc.)
	Self-ratability	Empowerment can be self-rated by patients
Round 2 & 3 Item: to express your opinion, thoughts or emotions	Relevance	Improvement on this item reflects improved digital health literacy of patients living with at least one chronic disease
	Improvability	Improvement on this item could result from interventions (*e.g.* training, etc.) during care
	Self-ratability	This item can be directly self-rated by patients

Group opinion was measured as percentages of agreement (%), and interquartile ranges (IQR) were calculated for round two responses. The percentage of agreement indicates the proportion of panelists who "agreed" or "strongly agreed" that each construct or item met the given assessment criteria. A higher percentage means that the statement was more widely endorsed by the group. Consensus attainment level was defined as strong (≥ 70% agreement), moderate (50–69%) or low (< 50%). It was not mandatory to write a comment, but the questionnaire emphasized that comments are crucial components for considering their opinion and improving the quality of choice of the PROM’s items. That way, experts’ comments were summarized and rephrased in a neutral manner to help orient the next round of consultation and to identify any major concern raised by the expert panel through the qualitative analysis [45].

Participants were invited by e-mail. Then, they received a survey link to an introduction page (Additional file 1). This page included information on the research team, the study, and a consent question, which were followed by a socio-demographic and clinical questionnaire. Personalized follow-ups were provided to panel members throughout the study, with a reminder sent to nonrespondents after two weeks during each round. Should there be no contact within the 2 weeks then no further communication was sent. Details on data collection and analysis are reported in the following paragraphs on a per-round basis. Qualitative data analyses were conducted using Word and Excel software (Microsoft Corp.), and statistical analyses were performed using Stata 14 (StataCorp LP).

### Round one: construct assessment

The first round aimed to assess consensus on the inclusion of 5 constructs in the PROM and their operational definitions (Table 3). Definitions of DHL and constructs were given to ensure that panelists would consider the DHL’s characteristics for patients in daily life. We also provided experts with the initial version of the PROM (14 items), so that they could link the constructs to the PROM’s items. Consensus for construct inclusion was defined as strong agreement (≥ 70%).

**Table 3:** Measurements constructs and definitions

Digital literacy	Operational knowledge in using everyday technological devices, such as computers and the internet
Reliability of information on internet	The degree of clarity in the process needed to select information
Relevance of information to personal health	Assessment of the relevance of information for decision-making based on individual differences (e.g. symptoms, medications, health promotion behaviors)
Privacy	The ability to make choices about the disclosure of personal information (including health data)
Empowerment	People's choices to interact with professionals “online” to improve have health-oriented behaviors

The results revealed that experts considered that the constructs were not well suited for self-rating, except for empowerment. Analysis of their comments led us to suspect that a potential misunderstanding of what was meant by self-ratability may have led to a systematic error in respondents’ ratings of this criterion. Therefore, it was clarified in the second-round survey: “Can the construct/item be directly evaluated by the patient, taking into account what the patient believes to be true (perception) and what he/she can do (with coping strategies leading to observable behavior)?” Finally, a major concern raised by the experts was the possibility that patients ask for help from someone else when using ICTs. Thus, we chose to incorporate this aspect directly into the concerned PROM’s questions to be assessed in round 2.

### Round two: assessment of items

In round two, experts assessed the PROM’s items (*n* = 14) to be included or excluded under each construct. At the beginning of the second-round questionnaire, they were invited to consider a summary of comments provided in round one. Interquartile ranges (IQR) were also calculated alongside percentages of agreement (%). The IQR refers to the dispersion of obtained ratings. A smaller range (low dispersion) indicates that the opinions of panelists were more consistent. Consensus for item inclusion was defined as strong agreement (≥ 70%) and low dispersion (IQR < 1) within the expert panel.

Many experts highlighted that the revised formulations of the PROM’s items incorporating the possibility to obtain help from someone else could be interpreted as double-barreled questions (DBQs). A DBQ is a question that asks about two or more issues but leaves a possibility for just a single answer. Basically, whenever respondents are force to answer two questions (disguised as one) with a single answer. So respondents may understand the stimuli in a DBQ differently, and answer based on one of them while disregarding the other. This can lead to an adverse effect on validity [46]. To avoid this, we opted to return to the original items’ formulations, as assessed in round one, during the final round but kept the ratings made by the experts in round two to decide whether these items should be included, excluded, or reassessed. An expert suggested that we look into the Health Assessment Questionnaire (HAQ) by B. Bruce & J.F. Fries [47] as an example to include a modality for considering the help of a person in a question. We used the question coming from the French adaptation of the HAQ Disability Index by Guillemin, Briancon & Pourel [48] to formalize the help of a person’s aspect by adding a separate question in the third round. A formal request for the use of the question was sent to the authors.

### Round three: final assessment of items

The third round aimed to assess revised consensus on 7 items that had initially obtained moderate or inconsistent agreement in the second round (Fig. 1) and one added question. Two items were reworded based on experts’ comments and one item was removed because it was seen as redundant with another. At the beginning of the third round, experts received individualized feedback on their round-two ratings, the position of the group for each item (Additional file 2) and a synthesis of comments. Participants were invited to consider the answers of the group to reassess their position and add final comments. Items were presented in descending order based on their percentage of agreement, as recommended in Delphi methodology [49].

### Data analysis

#### Quantitative data analysis

There is no definite criterion to determine consensus in Delphi studies. We choose a percentage agreement (> 70%) for all rounds and added a proportion within a specified range (IQR < 1) in round 2 (items assessment) to measure the level of consensus attainment [50, 51]. Descriptive statistics were performed to assess the convergence towards consensus (percentage, interquartile range, and number of comments) as suggested by von der Gracht [32]. A distributional analysis was done to assess the position of our panel regarding the evaluation of the three criteria for each construct or item.

Thus see Table 1, construct or items were considered acceptable for inclusion in the questionnaire if 70% of experts agreed or strongly agreed with the statement for all three criteria (relevance, improvability and self-ratability). A statement with an agreement between 50 and 69% was resubmitted in the next round. A statement with less than or equal to 49% agreement was rejected. For round two only we additionally considered that an IQR under 1.00 was required to indicate consensus for immediate inclusion.

#### Qualitative data analysis

Qualitative data were analyzed for each round according to the analysis method described by Miles, Hubermann & Sadaña [45], and organized by theme. Results of the analysis were presented using a conceptually clustered matrix charting participants’ comments about selected concepts [45]. All comments collected during the e-survey are presented in a way that remains close to the data provided (number of comments, inventory). This feature is crucial in presenting the results of a topical survey according to Sandelowski's typology [52]. From an application perspective, the comments were mapped to the Adaptation Model [23, 24] and the influencing factors that can affect coping strategies*: i)* personal (beliefs, values, genetics), *ii)* collective/group factors (physical facilities, financial resources, interpersonal relationships, social background and culture, decision-making and information systems), *iii)* technical (process, methods based on scientific knowledge, used in information management and decision making systems, *iv)* policy (health policy: context, infrastructure, evidence based nursing practice process and delivery systems) [53, 54]. The purpose of the qualitative analysis was to describe problems identified with the use of ICTs in healthcare, and the personal coping strategies used, as expressed by the panel.

According to the analysis method described by Miles & Hubermann [55], the first step is to use the clustering technique to define specific issues. The analyst identifies problems (or tensions) that underlie the comments. Then, a similar clustering is made in relation to "what can be done to solve the problem" and between these solutions as potential coping strategies. Then a conceptual sorting is carried out: is the proposed coping strategy of a personal, collective, technical, or political nature. The data entered were essentially short sentences (with the participant's code), and we used the double confirmation decision rule. We selected a similar proportion (> 50%) of comments from each round to double-code and analyze, ensuring that all DHL constructs were covered. A total of 81 comments (53%) were independently double-coded to ensure the reliability of the analyses. Then, inferences were directly made from the data presented: establishing patterns, themes and factoring (*i.e.* identifying a few general variables underlying many specific variables) which are illustrated by excerpts from the study comments. Full agreement between both researchers was required for inclusion of statements, with disagreements resolved through discussion [55]. Senior researchers reviewed the data at each stage for feedback and revision prior to dissemination. The presentation of the data was done using a conceptually clustered matrix (Additional file 3).

## Results

From the 42 experts invited, two were found to be ineligible (no consent given). The remaining 40 gave their consent to participate. Their characteristics are presented in Table 4. The majority of the panel experts were female (60%). Age was relatively balanced in the panel. Nursing represented the most common area of expertise (37.5%). Nine patient partners shared their expertise. Expert panel attrition was 10% during the second round (*n* = 4, 3 clinicians and 1 academic) and 5% during the third round (n = 2 clinicians). Thirty-four experts completed all e-Delphi rounds.

**Table 4:** Description of the panel of experts

First round (n = 40)		
Age, n (%)	25–34 35–44 45–54 55–64 65 +	- 5 (12.5) - 16 (40.0) - 8 (20.0) - 7 (17.5) - 4 (10.0)
Sex, n(%)	Men Women	- 16 (40.0) - 24 (60.0)
Principal occupation, n (%)	Academic Clinical Patient	- 14 (35.0) - 17 (42.5) - 9 (22.5)
Area of expertise, n (%)	Nursing Public health Medicine Patient (experience) e-Health Health literacy and/or DHL Geriatrics Other	- 15 (37.5) - 6 (15.0) - 4 (10.0) - 4 (10.0) - 4 (10.0) - 2 (5.0) - 1 (2.5) - 4 [10]

### Results round 1

During round one (*n* = 40 experts), five potentially important constructs were submitted. Table 5 summarizes the results of the Delphi process during each round. Based on the results obtained from the analysis of the qualitative and quantitative data, experts systematically raised concerns with the self-ratability nature of all constructs, except for *empowerment* (R- relevance: 87.2%, I = improvability: 92.3%, SR-self-ratability: 89.7%). SR’s percentage of agreement was 67.5% for *digital literacy*, 41% for *reliability of information on internet*, while *relevance of information to personal health* and *privacy* were both at 66.7%. The percentage of agreement about the relevance of the privacy construct was 69.2%, very close to our 70% threshold. We decided to keep it because it’s a relatively new construct in digital health literacy [15] and it represents a common preoccupation shared by experts. Interestingly, this construct is not present in health literacy studies [56, 57]. Finally, experts raised the need to account in the PROM for the possibility to ask someone for help when using technologies. The 5 constructs and 14 related items were proposed during the 2^nd^ round.

**Table 5:** Summary of results (R: relevance; I: improvability; SR: self-ratability; + : consensus achieved; ?: sent to next round; × : rejection)

Construct #. Items theme	Round 1			Round 2			Round 3			Finale decision
	R	I	SR	R	I	SR	R	I	RS	
***Digital literacy***	** + **	** + **	**?**							** + **
Use keyboard (*e.g.* to type words, phrases)				** + **	** + **	** + **				** + **
Using links on websites				** + **	** + **	** + **				** + **
Using a messaging system (*e.g.* writing an e-mail, instant message)				** + **	** + **	** + **				** + **
***Assessment of the reliability of information on internet***	** + **	** + **	** × **							** + **
Decide whether the information is reliable (based on research findings)				** + **	**?**	**?**				**x** Reworded
Check different websites to see if they provide the same information				** + **	**?**	**?**				**x** Reworded
Reworded for round 3: Find resources to check reliability of information (*e.g.* check different websites, ask health professionals)							** + **	** + **	**?**	** × **
Making a choice from all the information you find				** + **	**?**	**?**	** + **	** + **	** + **	** + **
***Assessment of the relevance of information to personal health***	** + **	** + **	**?**							** + **
Understanding information (*e.g.* simple vocabulary, short sentences, easy reading)				** + **	**?**	**?**	** + **	** + **	** + **	** + **
Apply the information you have found in your daily life				** + **	**?**	**?**				**x** Redundant with below
Use the information found to make decisions (*e.g.* on diet, physical activity, emotional well-being)				** + **	** + **	**?**	** + **	** + **	** + **	** + **
***Privacy***	**?**	** + **	**?**							** + **
Know who can read the message				** + **	** + **	**?**				**x** Reworded
Reworded for round 3: Knowing whether personal data protection standards are met							** + **	** + **	**?**	**x**
Share private information (*e.g.* your name, age, address)				** + **	**?**	** + **	** + **	** + **	** + **	** + **
Reworded in round 3: Do you feel confident sharing private information about your health online (*e.g.* diagnosis, medication, questions)?							** + **	**?**	** + **	**x**
***Empowerment***	** + **	** + **	** + **							** + **
Clearly state your question or health concern				** + **	** + **	** + **				** + **
Express your opinion, thoughts or emotions				** + **	** + **	** + **				** + **
Knowing which behaviors are good for your health				** + **	** + **	** + **				** + **
Introduced in round 3: Please indicate (tick) the activities for which you need help from someone (doctor, nurse, relative): and choice of suggestions							** + **	** + **	** + **	** + **

### Results round 2

Thirty-six panelists completed round two. During the second round, 6 items (3 within digital literacy and 3 within empowerment) achieved a consensus level of 70% or greater and were retained in the final PROM. No item was rejected outright during this round, but one item was removed as it was deemed redundant by experts. Three items were modified following experts’ comments: *decide whether the information is reliable* and *check different websites to see if they provide the same information* were merged into a single revised item; and *know who can read the message* was revised. The comments received confirmed that experts viewed *privacy* as an important construct. Furthermore, the notion of trust emerged linked to the issue of sharing health related information online to persons who are more or less known.

Seven items that did not reach consensus and one additional item [48] were reassessed in the final round.

### Results round 3

Thirty-four panelists assessed 8 items during the third round. Three items did not reach the acceptance threshold and were ultimately excluded from the PROM. The additional items accounting for external help from someone were accepted.

From our starting pool of 5 constructs and 14 items, 5 constructs and 11 items reached consensus to be included in the PROM. The 11-item PROM, named Lisan*e*, is currently only available in its original French version.

### Synthesis of qualitative results

The conceptually clustered matrix of results is in Additional file 3. Of the 180 comments made in the e-Delphi, 155 were usable (round 1 *n* = 44, round 2, *n* = 84, round 3, *n* = 27). Non usable comments were excluded because they were redundant on DBQs. The qualitative data extracted and analyzed [45] from the three round’s comments showed several problems arising from the utilization of ICTs. Firstly, most of the problems raised by the experts were formulated using minimal or no coping strategy. The most frequent problems identified related to the difficulty of accessing and using digital resources due to a lack of knowledge, understanding, skills or dexterity. Secondly, there was rarely a structural or organizational response highlighted by experts: the problems were largely treated as an individual learning effort with the support of the group or a technical support even if the possibility of adaptation with electronic devices may have limits (*e.g.*, related to age, cognitive disorders, understanding and critical thinking for use). Thirdly, there are two main realities underlying described problems. First is the ability to assess specific personal coping strategies and contextual characteristics (stimuli) of DHL that are modifiable. This would involve considering the answers rated as "difficult" or very "difficult" in the questionnaire and asking the patient: “For what reason(s)?” This would make it possible to adapt the way personal needs are discussed and to propose individualized intervention and TIC that is adapted to one’s digital literacy level. The second is to consider the digital tool as a contextual factor influencing the care of patients and their families within the health system.

## Discussion

This study used a e-Delphi approach to conduct a systematic process to assess consensus on the content of a new digital health literacy PROM. Three evaluation criteria, relevance, improvability and self-ratability, were used by experts to assess each item and construct. Over a three-round process, thirty-four panelists, including patient partners, clinicians and academics, reached consensus on 5 constructs and 11 items. This result reflected the multidimensional nature of DHL’s outcomes as described in previous work [26]. For health literacy measurement [58], the way DHL is understood should be closely linked to how it is measured. This means that measures need to follow the evolution of clinical practice with ICTs and chronic patient’s outcomes that are multiple [38, 59]. Furthermore, using a Delphi technique allowed us to determine which outcomes to measure in clinical practice, and for further research [60]. In our e-Delphi study, we captured academics, clinicians and patients’ comprehension of the characteristics of digital health literacy, which had substantial implications on the content validity of our new DHL PROM.

The instrument constructs that achieved a consensus among academics, clinicians and patients in this study were about digital literacy, reliability of information on the internet, relevance of information to personal health, privacy, and empowerment. These constructs are consistent with previous qualitative research investigating important aspects of chronic patients' skills when using ICT. A phenomenological study (*n* = 10) aimed at exploring the experience of using telemedicine with people with chronic obstructive pulmonary disease (COPD) identified several themes that are aligned with our consensus constructs, such as accessibility (health service), support from healthcare professionals (regular follow-up from nurses), enhanced clinical insight (e.g. daily self-measurement of clinical parameters), and mutual language (effective communication) [61]. An other study with a meta-ethnographic design (12 studies) targeting the experience of using telemedicine among patients living with COPD also identified constructs in concordance with our questionnaire. The synthesis revealed three first-order constructs and their second-order constructs: 1) presence: with accessibility, digital proximity; 2) transparency: with clinical awareness (an overview of patients' health status enabling greater awareness of their individual data), reciprocal dialogue (sharing clinical data and horizontality of clinical language); 3) ambivalence: independent but close (sense of security, control, dignity and independence), restricted but detached [62]. Empowerment more specifically is in line with the meta-analysis of Fernandes et al., 2022 who saw it as an important enabler for engaging in telehealth interventions [63]. Our findings reaffirm the prominent role of personal skills and empowerment for patients using ICT in health care. Moreover, our study highlighted that structural or organizational responses to problems arising from the use of ICTs were rarely considered, even if personal adaptation to electronic devices may have its limits. There are many barriers which can restrict the use of digital devices: infrastructure barriers (e.g. 4G available or not), financial barriers (e.g. an internet-connected smartphone), social attitude-exclusion (e.g. psychological issues), government support, and education, training and individual support [64–66]. It is important that resources are allocated to ensure that these barriers are removed so that people are able to access healthcare services.

We used an online survey strategy to recruit French-speaking experts knowledgeable in DHL for chronic patients. The e-Delphi method allowed patients to share their personal points of view about digital health literacy. Other methodologies can be used to obtain consensus on the constructs and items of a measure, including nominal groups techniques and focus groups. In-person methods can allow for richer discussions but are limited by the availability of the experts. Currently there is no best method to use for consensus [59].

The results of this study should be of interest to anyone seeking a better understanding of the measurement aspects of DHL in chronic patients, such as clinicians involved in e-health care environments. The measurement developed in this study is different from other validated measures of DHL because it identified relevant, improvable and self-ratable constructs as well as items including empowerment for chronic patients processing health information with electronic devices.

Further validation of the PROM will be required to consider issues regarding samples and setting in field-testing as recommended by Haynes, Richard & Kubany [67]. Since the PROM is self-completed, the usability and validity from the perspective of people presenting a wide range of literacy levels should be studied. According to evidence on existing measurement instruments of health literacy [19], we suggest that well developed instruments and validated instruments must be appropriately selected based on clinical practice.

### Strengths and limitations

This research project followed COSMIN recommendations for PROM development [21] and other recommendations to define outcomes criteria indicators [33, 34]. Limitations are to be noted. First, there were comparatively fewer patient than academic or clinical members in the panel. Although the Delphi process allowed patients’ comments to be fed back anonymously to potentially refine clinicians’ and academics’ opinions, patients may have had less weight on overall consensus in our study. Secondly, the change in focus of the assessment between the initial and subsequent rounds, carried out to introduce the experts gradually to the underlying constructs of DHL and the PROM, may have limited the number of iterations to assess the items. Two reworded items were ultimately rejected because only one of the three criteria turned out to be "uncertain". We cannot be sure that an additional round would have resolved this uncertainty in one way or another, but it remains a possibility.

The strengths of the study are the diversity of experts’ profile, and the low attrition rate between the three rounds of the e-Delphi. Patient partners play an important role in the health care system, but they are few and could not be representative of all patients. We used a structured and rigorous communication approach that circumvented important biases in group reasoning. Panelists submitted many rich comments and explanations to strengthen the group communication process. However, despite its numerous advantages, the e-Delphi method presents inherent challenges to patients with little or no digital health literacy. In addition to being limited in their access to online communication process, their understanding and opinions about the three evaluation criteria assessed in this study may have been very different from other panel members, thus increasing the difficulty of reaching consensus [68]. Nevertheless, their perspectives on the clarity and usability of the PROM are extremely relevant and should be explicitly sought out during the next stages of instrument validation.

### Implications for research

This multiple phase research project is working towards a new PROM for digital health literacy assessment of chronic patients who are facing problems associated to process health information from digital devices. Current measurement approaches report four main conceptual models and related measures to respect the dynamic context of DHL [27], without considering patient’s empowerment. Further research would benefit from assessing DHL anchored in person-centered frameworks [69] such as the Adaptation Model for an appropriate use in health care to ensure that any scale developed encompasses outcomes, values, and patients’ preferences to access health care. We have yet to evaluate the response process validity, and the internal and external validity of the proposed measure. To achieve this, it is essential to ensure that online administration of the questionnaire does not impede digitally disadvantaged groups from completing it.

## Conclusion

This study identified 5 DHL constructs and 11 items on which patients, clinicians, and academics agreed regarding their relevance, improvability, and self-ratability. In addition, we found that minimal or no coping strategies were expressed by experts in terms of difficulty to access and use digital resources. Structural or organizational responses were rarely highlighted, even though adaptation with electronic devices may have limits (*e.g.*, related to age). However, it is critical to consider DHL assessment instruments, their ease of deployment and the applications of their outputs in practice as we further integrate digital healthcare delivery. The resulting PROM will undergo further evaluation and may help the assessment of chronic patients’ abilities when using digital health.

### Supplementary Information

**Additional file 1.** e-Delphi_ survey introduction_ ENG

**Additional file 2.** MatrixRound-2_Feedback Participant Results

**Additional file 3.** Thematic Conceptual

## Data Availability

The data that support the findings of this study and the questionnaire Lisane are available from the corresponding author upon reasonable request.

## Abbreviations

COSMIN: COnsensus-based standards for the selection of health measurement instruments
DHL: Digital health literacy
*e*-Delphi: Electronic-Delphi
ICTs: Information and communications technology
PROM: Patient-reported outcome measure
REDCap: Research electronic data capture system
