# Supplementary material for: Development of a patient-reported outcome measure of digital health literacy for chronic patients: results of a French international online Delphi study
Source: BMC Nurs. 2023 Dec 14;22:476. doi: 10.1186/s12912-023-01633-x (PMC10720110; doi:10.1186/s12912-023-01633-x)
Supplement: Supplementary file 1 — Additional file 1. e-Delphi_ survey introduction_ ENG [file 12912_2023_1633_MOESM1_ESM.docx]

**e-Delphi_ survey introduction: page 1**

RED Cap* HOME PAGE

**Welcome message for the e-Delphi first round**

**Project title**: Lisane: Development and validation of the first version of an instrument to measure digital health literacy (DHL) in adults with at least one chronic illness.

The aim of the Lisane project is to develop the first version of an instrument for measuring and adapting to research into the skills needed by patients to make appropriate use of health information from digital resources in order to obtain the answers they need for their health (skills known as digital health literacy).

A preliminary instrument, **the Lisane questionnaire**, was developed on the basis of a systematic literature review of instruments for measuring digital health literacy and a qualitative study of patients and healthcare professionals with experience of using digital health resources (e.g. searching for health information via the Internet, using interactive health applications to communicate with other patients/professionals, messaging and making appointments online). The items (questions) presented were translated from English by the author. The final items will be back translated to ensure the overall quality of the translation.

**The research team**: Carole Délétroz, MSc Nursing, Université Laval, Quebec & HESAV, Switzerland. Marie-Pierre Gagnon, PhD, Université Laval, Quebec & Patrick Bodenmann, MD MSc, Université de Lausanne, Unisanté, Switzerland.

**Research funding**: This project is funded by doctoral scholarships from the Canada Research Chair in Health Technology and Practice, Université Laval, and with support from the Université de Lausanne - Université Laval partnership project.

Dear Sir or Madam

We are asking you to take part as an expert in an online Delphi consultation method to gather the opinions of a group of experts on a specific subject: **the Lisane questionnaire.** A version of the Lisane questionnaire can be downloaded at the bottom of this page.

Over the course of three survey rounds (i.e. three e-mailings), we will try to reach a consensus.

You have been identified for this study because you are (1) French-speaking and (2) because of your expertise.

The online Delphi study takes the form of questionnaires sent by e-mail.

The first questionnaire consists of evaluating whether each concept in the instrument is :

- Relevant for assessing digital health literacy in patients living with at least one chronic disease.

- Improvable (potentially modifiable) through interventions (e.g. training, coaching, support, changes to the environment, incentives, etc.).

- Self-ratable by patients.

If you volunteer to take part in this Delphi, we will ask you to carry out the following tasks:

- Read the document: Nature of the study and definition of concepts

- Read the document: Lisane questionnaire

- Complete the socio-demographic questionnaire and the questionnaire for the first consultation round within two weeks of receipt.

In mid-February 2022, you will receive the questionnaire for the second consultation round, which you must complete within two weeks of receiving it. Three to four weeks later, you will receive the questionnaire for the third consultation round, which you should also complete within two weeks of receiving it.

**Time commitment**

Your participation in the study will last as long as it takes you to read the information and consent, and to complete the questionnaires. This time is estimated at around 20 minutes per consultation round, for a total of approximately 60 minutes (3 rounds X 20 minutes). The Delphi is expected to last four months.

**Confidentiality**

Your identifying information will be kept confidential at all stages of the consultation. You will be identified by a unique number. The link between your identification number and your e-mail address will be kept in a secure document to which only the student investigator and her research supervisor will have access.

**Participants' rights**

Your participation is voluntary and you may withdraw from the study at any time.

Questions, comments or concerns?

The definition of concepts is available here: download the document.

The preliminary version of the Lisane questionnaire is available here: download the document.

Would you like to take part in the study?

YES

NO

**e-Delphi_ survey introduction: page 2**

First round Questionnaire

**********************************************************************************

As a reminder, the Lisane questionnaire is an instrument for measuring digital health literacy.

Definition of digital health literacy: Ability to search for, find, understand and evaluate health information from electronic resources, and to apply the knowledge acquired to tackle or resolve a health problem.

The Lisane questionnaire currently comprises five constructs: digital literacy; assessing the reliability of information on the Internet; relevance of information to personal health; confidentiality; and empowerment.

To answer the following questions, please keep the Lisane questionnaire in front of you.

For the five-construct proposed, please indicate your level of agreement with the three criteria **Relevance/Improvability/self-ratability.**

- Relevant for assessing digital health literacy in patients living with at least one chronic disease

- Improvable (potentially modifiable) through interventions (e.g. training, coaching, support, environmental modifications, incentives, etc.)

- Self-ratable by patients.

Note: *REDCap (**R**esearch **E**lectronic **D**ata **Cap**ture): secure web application for building and managing online surveys and databases
