# Supplementary material for: Development of a patient-reported outcome measure of digital health literacy for chronic patients: results of a French international online Delphi study
Source: BMC Nurs. 2023 Dec 14;22:476. doi: 10.1186/s12912-023-01633-x (PMC10720110; doi:10.1186/s12912-023-01633-x)
Supplement: Supplementary file 2 — Additional file 2. MatrixRound-2_Feedback Participant Results [file 12912_2023_1633_MOESM2_ESM.docx]

|  |  | Results  % and ÉIQ | Your position |
| --- | --- | --- | --- |
| ***Digital literacy*** |  |  |  |
| Use keyboard (e.g. to type words, phrases) | Relevance | 86.1% 1 [4-5] | 5 |
|  | Improvability | 75.0% 1 [4-5] | 2 |
|  | Self-ratability | 83.3% 1 [4-5] | 5 |
| Using links on websites | Relevance | 80.6% 1 [4-5] | 5 |
|  | Improvability | 75.0% 0 [4-4] | 2 |
|  | Self-ratability | 77.7% 0 [4-4] | 5 |
| Using a messaging system (e.g. writing an e-mail, instant message) | Relevance | 86.1% 1 [4-5] | 5 |
|  | Improvability | 80.6% 0 [4-4] | 2 |
|  | Self-ratability | 75.0% 1 [3-4] | 5 |
| ***Assessment of the reliability of information on internet*** |  |  |  |
| Decide whether the information is reliable (based on research findings) | Relevance | 83.3% 0 [4-4] | 4 |
|  | Improvability | 66.6% 1 [3-4] | 3 |
|  | Self-ratability | 50.0% 2 [2-4] | 4 |
| Check different websites to see if they provide the same information | Relevance | 75.0% 1 [4-5] | 4 |
|  | Improvability | 61.1% 1 [3-4] | 3 |
|  | Self-ratability | 63.9% 2 [2-4] | 4 |
| Making a choice from all the information you find | Relevance | 83.3% 1 [4-5] | 5 |
|  | Improvability | 69.5% 1 [3-4] | 2 |
|  | Self-ratability | 63.8% 1 [3-4] | 5 |
| ***Assessment of the relevance of information to personal health*** |  |  |  |
| Understanding information (e.g. simple vocabulary, short sentences, easy reading) | Relevance | 80.6% 1 [4-5] | 5 |
|  | Improvability | 58.3% 1 [3-4] | 2 |
|  | Self-ratability | 66.7% 2 [3-5] | 5 |
| Apply the information you have found in your daily life | Relevance | 80.5% 1 [4-5] | 5 |
|  | Improvability | 66.6% 1 [3-4] | 2 |
|  | Self-ratability | 66.6% 1 [3-5] | 5 |
| Use the information found to make decisions (e.g. on diet, physical activity, emotional well-being) | Relevance | 83.3% 1 [4-5] | 5 |
|  | Improvability | 77.7% 0 [4-4] | 2 |
|  | Self-ratability | 58.3% 2 [2-4] | 5 |
| ***Privacy*** |  |  |  |
| Know who can read the message | Relevance | 72.3% 2 [3-5] | 5 |
|  | Improvability | 72.2% 1 [3-4] | 1 |
|  | Self-ratability | 58.4% 2 [3-5] | 5 |
| Share private information (e.g. your name, age, address) | Relevance | 74.3% 2 [3-5] | 4 |
|  | Improvability | 65.7% 1 [3-4] | 2 |
|  | Self-ratability | 74.3% 1 [4-5] | 4 |
| ***Empowerment*** |  |  |  |
| Clearly state your question or health concern | Relevance | 85.3% 1 [4-5] | 5 |
|  | Improvability | 79.4% 1 [4-5] | 2 |
|  | Self-ratability | 85.3% 1 [4-5] | 5 |
| Express your opinion, thoughts or emotions | Relevance | 76.5% 1 [4-5] | 5 |
|  | Improvability | 76.5% 0 [4-4] | 2 |
|  | Self-ratability | 82.4% 1 [4-5] | 5 |
| Knowing which behaviours are good for your health | Relevance | 75.8% 1 [4-5] | 5 |
|  | Improvability | 81.8% 1 [3-4] | 2 |
