# Supplementary material for: Development of a patient-reported outcome measure of digital health literacy for chronic patients: results of a French international online Delphi study
Source: BMC Nurs. 2023 Dec 14;22:476. doi: 10.1186/s12912-023-01633-x (PMC10720110; doi:10.1186/s12912-023-01633-x)
Supplement: Supplementary file 3 — Additional file 3. Thematic Conceptual [file 12912_2023_1633_MOESM3_ESM.docx]

**Thematic conceptual matrix: problem and coping strategy (Miles, 2020, pp.168-171)**

| **Problems** | **Coping strategies (potential means)** | | | |
| --- | --- | --- | --- | --- |
|  | Personal : beliefs, values, genetics (influencing factor) | Group  (influencing factor) | Technical (influencing factor) | Policy: organisation of health care and services (influencing factor) |
| **Digital literacy :** Access AND use AND  Evaluation/self-assessment | **Adaptation (flexibility) :**  taking into account limitations, e.g. elderly, people with cognitive impairments, low literacy | **Training :**  acquisition of skills for practical explanations  **Family and friends, help desk :**  help and support  **Communication :**  Digital tools to communicate with others | **Capacity :**  no or little use of digital tools  **Understanding of terms :**  specific words (jargon). E.g. choice of words, definitions: digital, numerical ? | **Access to digital tools** ( computer / phone)  **Speed :**  Not all people using digital tools are fast digital development  (no help by the health system)  **Digital divide** |
| **Evaluation of information on the Internet :**  Complexity to stand back from information AND  Critical thinking required | **Cognitive skills**  **:**  understanding of language, self-perception, ability to stand back (e.g. analysis of information during the Covid 19 pandemic)  **Adaptation (flexibility)** taking limitations into account  **Demonstrate objectivity** | **Training/skills :**  critically review information found; compare information on several websites  **Relatives, help desk :** help and support | **Information overload :**  large number of sites  **Disinformation :**  fake news  **Internet environment** **:** cyber risks and cyber security | **e-Health interventions :**  listing reliable, credible and objective information on several websites.  **Social network** |
| **The relevance of information to personal health :**  Complexity of adapting general information to a particular situation | **Motivation :**  is a positive resource for information seeking  **Specific situation**  (e.g. presence of symptoms, disease)  **Capacity for self-assessment :**  relevance of information to one's situation is difficult to know  **Hope, beliefs about the disease  :**  belief that everything is relevant may be present | **Person's skills :**  identifying the source of information  **Education :**  literacy level  **Literacy level:**  Increase written information with simple and clear vocabulary  **Understanding :**  have explicit links between information | **Health behaviours   :**  physical activity with connected watch as an additional motivation | **Standards :**  Are pathology and medication not health literacy specifically |

| **Privacy :**  Sensitive to the promotion of literacy and the  “digital interface” | **Choice of digital tools :**  used by the person  **Digital knowledge   :**  level of the person’s knowledge | **Hacking :**  computer targeting, or any other actions that target personal data  **Knowledge :**  the impact of a lack of confidentiality in health | **Patients collaboration :**  digital management of their data  **Meeting with the Dr**  **:**  passing on sensitive information, trust and guaranteed confidentiality | **Laws :**  Knowledge of data protection (RGPD)  **Standards :**  Privacy is not explicitly part of the definition of health literacy |
| --- | --- | --- | --- | --- |
| **Empowerment :**  Not only action, but also awareness and critical thinking about environments | **Motivation :**  enables one to act on one's choices about one's health  **Ability to formulate questions :**  face to face, as well as remotely via video-conference  **Choice of therapies and objective :**  traditional medicine and other approaches : homeopathy, osteopathy, meditation, stress reduction, etc. | **Institution :**  To simplify messages  **Training :** at the individual level and associated contextual factors  **Consider other factors :**  available resources, social network in addition to information  **Ensuring a form of autonomy :** to help the person to develop a critical mind.  **Reading patient record by health professionals :**  importance of having information about patient | **How to use :** telehealth, chat-bot | **Structure of health system :**  Patients’thougths on interventions so as to relieve the care structure  **Pandemic :** confinement and fear of the risks. This aspect is important if not determinant factor.  **Empowerment :**  is part of current definitions of health literacy. It is not limited to individuals’action |

**Legend**

Academics

Clinicians

Patients
